# Dietary Energy Sources Affect Cecal and Fecal Microbiota of Healthy Horses

**DOI:** 10.3390/ani14233494

**Published:** 2024-12-03

**Authors:** Laura A. Brandi, Alanne T. Nunes, Camila A. Faleiros, Mirele D. Poleti, Elisângela C. de M. Oliveira, Natalia T. Schmidt, Ricardo L. M. Sousa, Heidge Fukumasu, Julio C. C. Balieiro, Roberta A. Brandi

**Affiliations:** 1 Department of Animal Science, School of Animal Science and Food Engineering (FZEA), University of São Paulo (USP), Pirassununga 13635-900, São Paulo, Brazil; lauraalvesb1@gmail.com (L.A.B.); nataliaschmidt@hotmail.com (N.T.S.); 2 Department of Veterinary Medicine, School of Animal Science and Food Engineering (FZEA), University of São Paulo (USP), Pirassununga 13635-900, São Paulo, Brazil; alanne.nunes@usp.br (A.T.N.); camilafaleiros@usp.br (C.A.F.); mirelep@usp.br (M.D.P.); limattos@usp.br (E.C.d.M.O.); rlmoros@usp.br (R.L.M.S.); fukumasu@usp.br (H.F.); 3 Department of Animal Nutrition and Production, School of Veterinary Medicine and Animal Science (FMVZ), University of São Paulo (USP), Pirassununga 13635-900, São Paulo, Brazil; balieiro@usp.br

**Keywords:** bacteria, concentrate, horse, hay, oil

## Abstract

Different energy sources are often used in horse diets to enhance health and performance. Understanding how diet impacts the cecal and fecal microbiota is crucial for meeting the nutritional needs of horses. High-throughput sequencing and qPCR were used to compare the fecal and cecal microbiota of five healthy horses receiving three different diets: hay diet (HAY), hay + starch and sugar (SS), and hay + fiber and oil ingredients (FO). Assessment of short-chain fatty acids, pH, and buffer capacity was also performed. The HAY diet was associated with the highest values of fecal pH; the FO and SS diets were associated with higher values of BC6 in the cecum, and the SS diet had higher BC5 values in feces (*p* < 0.05). HAY was associated with a lower alpha diversity in feces and with a higher abundance of Treponema, Fibrobacter, Lachnospiraceae AC2044, and Prevotellaceae UCG-003 in feces. SS was associated with a higher abundance of Desulfovibrio, the Lachnospiraceae AC2044 group, and Streptococcus in the cecum, and Streptococcus and Prevotellaceae UCG-001 in feces, while FO was associated with higher Prevotella, Prevotellaceae UCG-003, and Akkermansia in the cecum, and the Rikenellaceae RC9 gut group and Ruminococcus in feces. This study indicated that different energy sources can influence cecal and fecal microbiota composition and fecal diversity without significantly affecting fermentation processes under experimental conditions. These findings suggest that the diets studied may not pose immediate health risks; however, further research is needed to generalize these effects on gastrointestinal microbiota in broader equine populations.

## Simple Summary

The fecal and cecal microbiomes of healthy horses fed a HAY diet, hay + starch and sugar (SS), and hay + fiber and oil ingredients (FO) were compared using high-throughput sequencing and qPCR. Diet effects were also compared by assessing short-chain fatty acids, pH, and buffer capacity (BC). HAY was associated with lower alpha diversity in feces and a higher abundance of fiber-degrading bacterial genera. In contrast, SS showed a higher abundance of genera linked to non-structural carbohydrate fermentation and intestinal acidosis, while FO was associated with genera related to fiber degradation and markers of improved health status. The highest values of fecal pH were observed in HAY; higher values of BC at pH 6 were observed in the cecum in FO and SS, and a higher BC at pH 5 was observed in the feces of SS. Taken together, these data demonstrated that diets based on fiber, SS, and FO, although influencing cecal and fecal microbiota and fecal diversity, did not significantly affect digestive parameters and might not promote health risks to horses.

## 1. Introduction

Fiber-based diets are fundamental for horse nutrition, ensuring both maintenance requirements and intestinal health [1]. However, to enhance the productive performance of horses, there has been an increasing trend toward energy supplementation through the inclusion of grains [2], which can result in starch overload and disruption of the gut microbiota [3,4]. Oils, as energy-dense dietary fat sources, are promising alternatives for boosting dietary energy content without compromising intestinal health [5,6].

Diet strongly influences the hindgut environment, which is crucial for equine health [7,8]. Variations in dietary energy sources impact the microbial fermentation of carbohydrates into energy-yielding products such as short-chain fatty acids (SCFAs) [3] that supply up to 70% of the energy in equines [9] and affect the intestinal pH and buffering capacity, critical factors for digestive health [10]. Although commonly used to meet the high energy demands of horses, high-grain diets can lead to significant fluctuations in hindgut pH and SCFA concentrations [11,12,13]. In terms of microbial populations, these diets increase the presence of non-structural carbohydrate-fermenting bacteria, such as Lactobacillaceae and Streptococcus [3,11,14]. This elevation can result in higher lactic acid production and an increased risk of acidosis [15]. Additionally, starch overload can reduce bacterial richness and diversity, diminishing resilience and increasing the susceptibility to dysbiosis [2,11,16].

Oil supplementation has emerged as a viable alternative for increasing dietary energy while reducing reliance on grain [17]. Previous studies have demonstrated the physiological benefits of oil supplementation in horses, such as a decreased thermal load and enhanced metabolic adaptations, along with the provision of essential fatty acids [18]. Despite these benefits, the effects of oil supplementation on the intestinal microbiota, particularly within specific gut compartments, remain underexplored. Incorporating non-starch energy sources into equine diets may maintain digestive parameters and microbiota profiles more closely aligned with those observed in forage-based diets. Thus, the objective of this study was to evaluate the effects of different energy sources on digestive parameters and the cecal and fecal microbiota of healthy horses. This comprehensive approach ensures that the findings can inform practical dietary adjustments that support both the performance and well-being of horses.

## 2. Materials and Methods

### 2.1. Ethics Statement

All experimental procedures related to animal management and sampling described below were approved by the Animal Research Ethics Committee (CEUA) at the School of Agricultural and Veterinary Sciences (FCAV), São Paulo State University (UNESP), Brazil (protocol No. 2057/21).

### 2.2. Experimental Design and Sample Collection

Five healthy mixed-breed horses of both sexes, aged 10 ± 2.6 years and weighing 384 ± 9.51 kg, were used in this experiment. Horses were housed in individual stalls equipped with an automatic drinker and feeder. The animals had access to a fenced area with water and without pasture for 4 h a day (2 h in the morning and 2 h in the afternoon). However, throughout the experiment, there were losses of access to the cecum and the mortality of one animal. The description of the number of animals evaluated at each stage of the experiment is provided in the footnotes of the tables and figures.

Treatments comprised three diets, as follows: HAY diet consisting exclusively of Tifton-85 hay (*Cynodon* spp.), starch and sugar (SS) diet, and fiber and oil (FO) diet. Diets were formulated to meet the nutritional requirements of horses in maintenance according to National Research Council recommendations [1]. Horses assigned to the HAY diet received 8.4 ± 1.36 kg of Tifton-85 hay daily (dry matter, DM, basis), and those assigned to the SS diet received 5.38 ± 0.15 kg of Tifton-85 hay and 2.6 ± 0.07 kg of concentrate (SS base) daily. The SS diet concentrate included ground corn, soybean meal, wheat meal, rice meal, corn gluten meal (21% protein), *Chlorella vulgaris* Algae flour, inactive yeast, ground rice hulls, sodium chloride, cane molasses, calcite limestone, calcium carbonate, L-Lysine, DL-Methionine, and vitamin-mineral supplement (mineral salt). Those assigned to the FO diet received 4.28 ± 2.39 kg of Tifton-85 hay and 1.9 ± 0.00 kg of concentrate (FO base) plus 300 mL of soybean oil daily. The FO diet concentrate included alfalfa hay, corn gluten meal (21% protein), ground soybean hulls, ground rice hulls, soybean oil, soybean meal, wheat meal, *Chlorella vulgaris* Algae flour, inactive yeast, cane molasses, sodium chloride, calcite limestone, calcium carbonate, L-Lysine, DL-Methionine, and the same vitamin-mineral supplement.

Bromatological analyses were conducted at the School of Veterinary Medicine and Animal Science (University of São Paulo) (Table 1). The content of DM, ash, ether extract, crude protein, and crude energy was determined following protocols outlined by the Association of Official Analytical Chemists [19]. For fiber analysis, crude fiber, neutral detergent fiber, acid detergent fiber, and hemicellulose were quantified according to the methods of Van Soest et al. [20]. Starch content was assessed through enzymatic degradation using Termamyl Amyloglucosidase AMG 300 L (Novozymes, Bagsvaerd, Denmark), with absorbance measurements taken via a spectrophotometer (SBA 200, CELM), as specified by Hendrix [21]. Diets containing concentrate (SS and FO diets) had a roughage/concentrate ratio of 70:30 on a DM basis and 56:44 on an energy basis. Diets did not exceed the maximum starch content of 1.1 g kg^−1^ live weight per feeding occasion [22]. Furthermore, we attempted to maintain protein and energy levels as similar as possible between the diets. All diets supplied the minimum recommended amount of 30% neutral detergent fiber [23]. Intake per meal and per kilogram of body weight is provided in Supplementary Materials Table S1.

**Figure 1:**
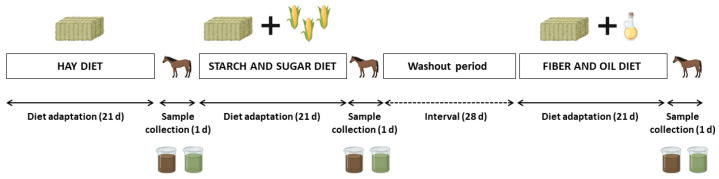
Experimental design with diet adaptation and sample (cecal fluid and feces) collection periods for analysis of the intestinal microbiota.

A cross-over design was used. Each experimental period comprised 21 days of diet adaptation, followed by one day of sample collection (Figure 1). All the animals were fed three different diets, starting with the HAY diet, then the SS diet, and lastly, the FO diet. The interval (washout) between the SS and FO diets was 28 days to avoid carryover effects. During this period, horses were released in a fenced area during the day and brought back to the stalls at night, where they had *ad libitum* access to Tifton-85 hay (*Cynodon* spp.) and mineral salt (50 g day^−1^).

For cecal fluid sampling, horses were subjected to cecal cannulation following a method adapted from Uribe Diaz et al. [24] and Kristoffersen et al. [3]. Briefly, after a prior typhlopexy procedure (fixation of the cecum to the abdominal wall), acepromazine maleate (0.05 mg/kg, IV) was administered, followed 15 min later by xylazine hydrochloride (0.3 mg/kg, IV). Once the bandage was removed, the skin near the incision site was cleansed with a 0.9% sodium chloride solution and neutral soap. Antiseptic preparation was then performed on the exposed serosa of the cecum. Local infiltration anesthesia was applied with 2% lidocaine hydrochloride (20 mL per animal) around the edges of the surgical site. A fenestration was then made in the previously fixed cecum via a circular incision approximately 2 cm in diameter, resecting a segment of the cecal wall. Fecal samples were collected directly from the rectum, according to Grimm et al. [25]. For microbial analysis, 10 mL of cecal fluid and 10 g of feces were collected on the sixth day of each experimental period and separately aliquoted into 5 mL cryotubes and stored at −80 °C in liquid nitrogen. For SCFA, pH, and buffer capacity analysis, 10 mL of the cecal fluid and 50 g of feces were collected on the sixth day of each experimental period. To prepare the fecal sample, 50 g of feces was mixed with 100 mL of distilled water, thoroughly homogenized, and then filtered. Cecal and fecal samples were stored in Eppendorf^®^ and Falcon tubes, respectively, and kept at −20 °C until further analysis.

### 2.3. Phenotypes

To determine digestive parameters (SCFA, pH, and buffer capacity at pH6 and pH5), samples were prepared according to the methodology described by Hussein et al. [26], and the concentrations of SCFA, acetate, propionate, and butyrate were measured in the Ruminal Fermentability Laboratory of the Faculty of Zootechnics and Food Engineering of the University of São Paulo (Pirassununga, Brazil) using gas chromatography (Shimadzu Corporation, Kyoto, Japan). To analyze the concentration of SCFAs in cecal fluid and feces, the methodology adapted by Warzecha et al. [4] was used. The pH determination was carried out using a portable pH meter according to the methodology of Santos et al. [10]. The buffering capacity at pH6 (BC6) and pH5 (BC5) was measured according to the methodology adapted from Zeyner et al. [27]. For BC in the cecum, 50 mL of cecal fluid was filtered through 15 cm filter paper, and 50 mL of the sample was obtained as a final solution. For BC in feces, 50 g of feces was homogenized and filtered through 15 cm of filter paper, removing 80 mL of the fecal sample solution. The final solutions of the cecal fluid and fecal samples were titrated with 0.25 M acetic acid, noting the volume of acid used when the pH reached 6 and pH 5 to determine BC6 and BC5, respectively. To calculate the BC, the formula was BC (mmol/L) = Volume (mL) × 3.125.

### 2.4. 16S rRNA Sequencing

Microbiome DNA was extracted with the GenElute Stool DNA Isolation kit (Sigma–Aldrich, Darmstadt, Germany), following the manufacturer’s recommendations. The DNA concentration and purity were evaluated using a Nanodrop One/One spectrophotometer (Thermo Fisher Scientific, Inc., Waltham, MA, USA). The DNA library was constructed according to Illumina recommendations (San Diego, CA, USA).

The first PCR was performed for locus-specific amplification of the hypervariable V4 region of the 16S rRNA gene [28,29] using primers 16S-515F (5′-GTGYCAGCMGCCGCGGTAA-3′) and 16S-806R (5′-GGACTACNVGGGTWTCTAAT-3′). For subsequent index PCR, Illumina sequencing adapters were added to the amplicon targets using Nextera XT Index Kit (Illumina, San Diego, CA, USA). The concentration of the PCR products was measured using Qubit 3.0 Thermo Fisher Scientific, Inc., Waltham, MA, USA). After quantification, the amplicon was pooled in equimolar proportions from the libraries, and pair-end 2 × 150 bp sequencing was performed using an Illumina MiSeq platform with MiSeq Reagent kit v2 (300 cycles).

### 2.5. Bioinformatics Analysis

Demultiplexed sequencing read files were retrieved from the Illumina BaseSpace^®^ website (https://platform.login.illumina.com/platform-services-manager, accessed on 8 May 2023). The read quality was checked with FastQC software2 (version 0.12.0), taking into account a quality score greater than 30. The 16S rRNA gene sequence data were analyzed using DADA2 package version 1.24.0 of R software version 4.2.3 [30] to determine the sequences into Amplicon Sequence Variants (ASVs). The ASV pipelines typically exhibit enhanced sensitivity, specificity, and precision compared to OTU algorithms. Additionally, they tend to have lower rates of spurious sequences and facilities for the integration of biological features across studies [30].

Briefly, forward and reverse reads were merged after denoising, chimeric sequences were removed, and taxonomy was assigned to the resulting amplicon sequence variants (ASVs) using SILVA database version 138.1. ASVs with less than 5 counts were not included in the analysis for improved accuracy of sequence readings and reduced bias of possible sequence errors [31]. A total of more than 20,000 sequences per sample were used for normalization of sequence readings [32]. Further analysis of the ASV table was performed using the MicroEco package (Version 1.9.2) [33] and phyloseq package (Version 1.38) [34] in R software version 4.2.3. Microbial diversity and richness were analyzed using the Shannon and Chao1 indices, respectively.

### 2.6. Quantification of Archaea and Protozoa

The quantification of archaea and protozoa populations in the samples was carried out using the relative quantification methodology described by Denman and McSweeney [35], which uses the total bacteria primer set as a housekeeping gene for normalization of the data. Real-time quantitative PCR was performed on a Bio-Rad CFX96TM Touch system (Bio-Rad Laboratories, Inc., Hercules, CA, USA). The primer sequences used are described in Table 2. Each sample had a final volume of 10 µL per well containing 2× qPCRBIO SyGreen Mix (PCR Biosystems, London, UK), 25 ng/µL of DNA, 300 nM of primer (archaea and total bacteria) or 100 nM primer (protozoa). A negative control was included in each plate to detect potential contamination or primer dimerization. Cycling conditions for archaea and total bacteria were as follows: 95 °C for 20 s, followed by 40 cycles of 95 °C for 3 s, 60 °C for 30 s, and 79 °C for 10 s. Cycling conditions for protozoa were as follows: 95 °C for 20 s, followed by 40 cycles of 95 °C for 3 s and 60 °C for 30 s. A melting curve was constructed immediately after amplification to check amplicon specificity. Fluorescence detection was performed at the end of each denaturation and extension step. Relative quantification of archaeal and protozoan populations was calculated using the ratio of Ct values of target genes to Ct values for total bacteria.

### 2.7. Statistical Analysis

A cross-over design was used in this study. For microbiota, SCFA, pH, and buffering capacity analysis, a generalized linear mixed model was used, with the fixed effect of diet and the random effect of the animal. The random effect of the animal was considered a variation factor, and repeated measures within each animal were considered evaluation conditions, reflecting the within-animal correlation structure. For microbial taxon, it was assumed that abundance followed a binomial distribution. A logistic linkage function was used to relate the observed abundances to the systematic components of the statistical model. The statistical model accounted for the fixed effect of diet and the random effect of the animal. Shannon and Chao1 indices were used for diversity analysis.

Different covariance structures were evaluated, and the best model was selected based on the Akaike information criterion [38]. In the case of a significant *F*-statistic for the source of dietary variation, Tukey’s test was applied to compare means at the 5% significance level. All analyses were performed using the PROC MIXED and GLIMMIX procedures of SAS version 9.4 (SAS Institute Inc., Cary, NC, USA, 2013).

## 3. Results

### 3.1. Phenotypes

There were no effects of diet on the concentration of total SCFAs, acetate, butyrate, and propionate), or the ratio of (acetate/butyrate)/propionate in the cecum and fecal samples (*p* > 0.05) (Table 3). The molar percentages of individual SCFAs are presented in Supplementary Materials Table S2.

Dietary treatments did not affect cecal pH (*p* > 0.05); however, there was an effect (*p* < 0.05) on fecal pH, with the HAY diet showing the highest values. For BC, no diet effect was observed for BC5 in the cecum (*p* > 0.05), but there was an effect on BC6 (*p* < 0.05), with the highest values observed in the SS and FO diet. In feces, diet did not affect BC6 (*p* > 0.05), but the SS diet resulted in a higher BC5 (*p* < 0.05) (Table 4).

### 3.2. Microbiome Composition Analysis

A total of 3,351,219 reads were generated for all samples (cecum + feces) from sequencing. After filtration, 2,452,067 reads were obtained, assigned to 8307 kingdoms, 7947 phyla, 7842 classes, 7505 orders, 5808 families, 3094 genera, and 1 species. The basic quality controls performed revealed high-quality reads and a mean of 90,368 +/− 9442 reads for samples. The rarefaction curves for microbial populations in the cecal and fecal samples reached the plateau, indicating sequencing depth was sufficient to describe the biodiversity within the dataset (Supplementary Materials Figure S1).

**Figure 2:**
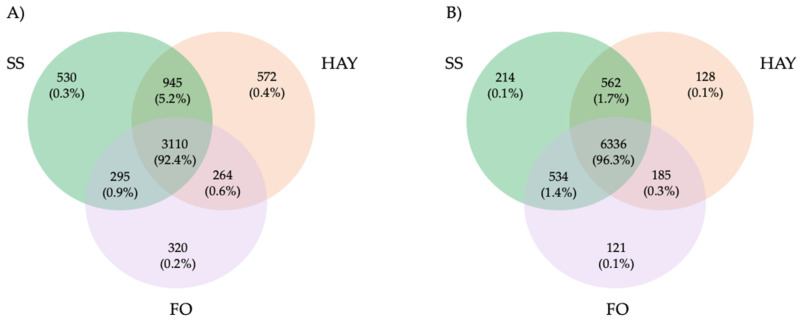
Taxonomic composition of cecum (**A**) and fecal (**B**) samples of healthy horses. Venn diagrams representing the core unique and shared microbiomes of horses supplemented with hay, starch and sugar (SS), and fiber and oil (FO) diets.

In fecal samples, a total of 8080 ASVs were detected, of which 562 (1.7%) were present in both the SS and HAY diets, 185 (0.3%) in the HAY and FO diets, and 534 (1.4%) in the SS and FO diets. A total of 6336 (96.3%) were detected in all the treatments. For cecal samples, a total of 6036 ASVs were detected, of which 945 (5.2%) were present in both the SS and HAY diets, 264 (0.6%) in the HAY and FO diets, and 295 (0.9%) in the SS and FO diets. A total of 3110 (92.4%) were detected in all the treatments (Figure 2).

### 3.3. Alpha and Beta Diversity

A comparison of the alpha diversity among diets was conducted to analyze the microbial species richness and diversity among diets (Table 5). Cecal samples revealed no significant difference in the Chao and Shannon index. However, the Shannon index was lower in fecal samples of the horses fed the HAY compared to the SS diet (*p* < 0.05).

**Figure 3:**
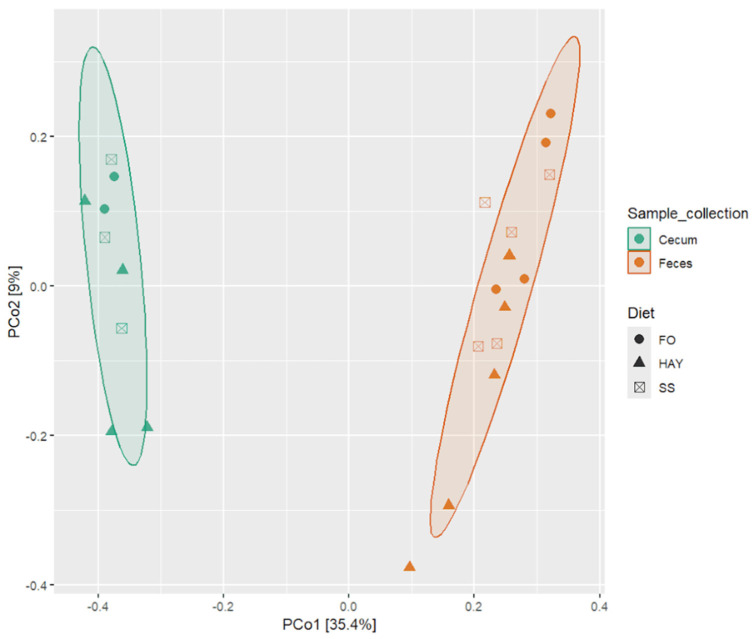
Principal Coordinate Analysis (PCoA) based on the Bray–Curtis distance for the analysis of the effect of the FO, HAY, and SS diets on bacterial microbiota in the cecum and feces samples from healthy horses.

To evaluate the impact of the diets on microbiota composition across different sample collection sites, we conducted pairwise PERMANOVA analysis using the Bray–Curtis distance matrix, considering all detected ASVs to assess beta diversity. Significant differences in microbiota were observed across the sampled site (*p* < 0.05), while it was observed a tendency (*p* < 0.10) when comparing the microbiota for the different diets within each sampled site, which can be visualized through Principal Coordinate Analysis (PCoA) based on the Bray–Curtis distance matrix (Figure 3).

### 3.4. Analysis of the Bacterial Community Composition at the Phylum Level

**Figure 4:**
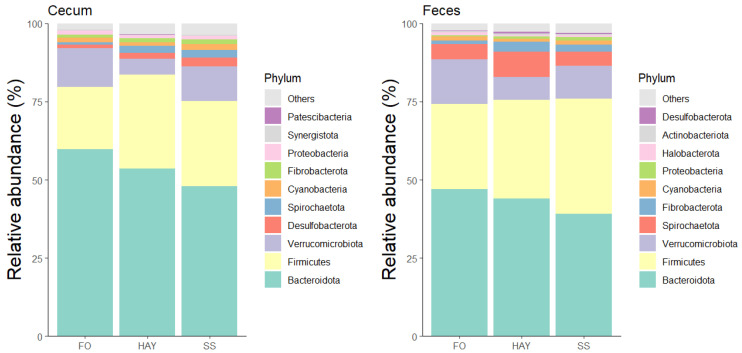
Relative abundance of the 10 major bacterial phyla in cecum and feces of healthy horses fed diets based on hay, starch and sugar (SS), and fiber and oil (FO). Total of samples: HAY (5 fecal, 4 cecal), SS (5 fecal, 3 cecal), FO (4 fecal, 2 cecal); variations due to cecum access loss and one mortality.

The 10 most bacterial phyla identified in the cecal and fecal samples are represented in Figure 4. In the cecal samples, the relative abundance of Firmicutes and Fibrobactera was similar between the HAY and SS diets (*p* ≥ 0.05) but higher compared to the FO diet (*p* < 0.05). The relative abundance of Desulfobacterota and Spirochaetota was highest in the SS diet, intermediate in the HAY diet, and lowest in the FO diet (*p* < 0.05). In fecal samples, there was a greater abundance of Spirochaetota and Fibrobacterota in the HAY diet (*p* < 0.05), while the relative abundance of Firmicutes was higher in the SS diet. The relative abundance of Bacteirodota and Verrucomicrobiota was highest in the FO diet for cecal and fecal samples (*p* < 0.05).

### 3.5. Analysis of the Bacterial Community Composition at the Family Level

**Figure 5:**
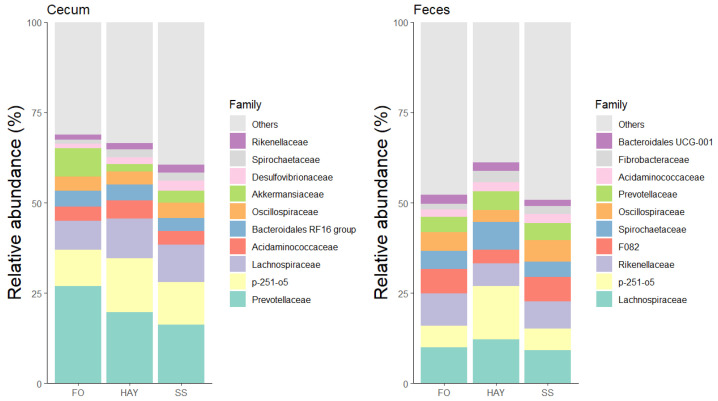
Relative abundance of the 10 major bacterial families in cecum and feces of healthy horses fed diets based on hay, starch and sugar (SS), and fiber and oil (FO). Total of samples: HAY (5 fecal, 4 cecal), SS (5 fecal, 3 cecal), FO (4 fecal, 2 cecal); variations due to cecum access loss and one mortality.

The 10 most bacterial families identified in the cecal and fecal samples are represented in Figure 5. In cecal samples, the relative abundance of Acidaminococcaceae and Ruminococcaceae was higher in the HAY diet, while Lachnospiraceae, Desulfovibrionaceae, and Spirochaetaceae were higher in the SS diet, and Prevotellaceae in the FO diet (*p* < 0.05). The relative abundance of Rikenellaceae was similar between the HAY and SS diets and between the HAY and FO diets (*p* ≥ 0.05), while the relative abundance of Fibrobacteraceae was lowest in the FO diet compared to the other treatments (*p* < 0.05). In fecal samples, the relative abundance of Spirochaetaceae, Lachnospiraceae, Selenomonadaceae, and Fibrobacteraceae was higher in the HAY diet, while the relative abundance of Ruminococcaceae and Rikenellaceae was higher in the FO diet. The relative abundance of Acidaminococcaceae was similar between the HAY and SS diets (*p* ≥ 0.05) but higher compared to the FO diet (*p* < 0.05).

### 3.6. Analysis of the Bacterial Community Composition at the Genus Level

**Figure 6:**
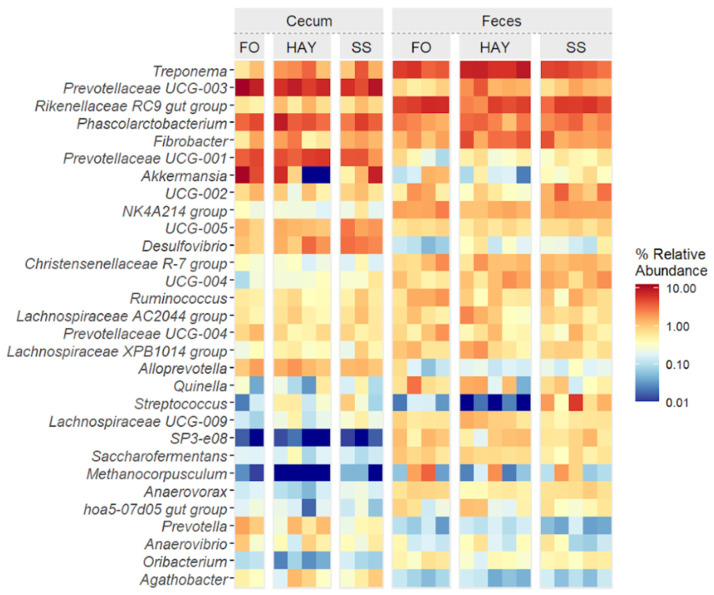
Heatmap of the top 30 bacterial genera in cecum and feces of healthy horses fed diets based on hay, starch and sugar (SS), and fiber and oil (FO). Total of samples: HAY (5 fecal, 4 cecal), SS (5 fecal, 3 cecal), FO (4 fecal, 2 cecal); variations due to cecum access loss and one mortality.

The top 30 bacterial genera identified in cecal and fecal samples are represented in Figure 6. Cluster analysis at the lowest taxonomic level identified distinct groupings among the most prevalent ASVs. A clear distinction was observed between cecum and fecal samples, along with notable diet-induced variations in the relative abundance of specific genera at each sampling site.

In cecal samples, a greater relative abundance of Treponema, Desulfovibrio, and the Lachnospiraceae AC2044 group was observed in the SS diet (*p* < 0.05). The relative abundance of Fibrobacter and Rikenellaceae RC9 gut groups was similar between the HAY and SS diets (*p* ≥ 0.05) but higher compared to the FO diet (*p* < 0.05). The relative abundance of Prevotellaceae UCG-004 and Anaerovibrio was similar between the HAY and SS diets (*p* ≥ 0.05) but lower compared to FO (*p* < 0.05). The FO diet showed the highest relative abundance of Prevotella, Prevotellaceae UCG-003, and Akkermansia (*p* < 0.05). The relative abundance of Prevotellaceae UCG-001 was similar between the HAY and FO diets (*p* ≥ 0.05) but higher compared to the SS diet (*p* < 0.05). In fecal samples, the SS diet showed the highest relative abundance of Streptococcus and Prevotellaceae UCG-001 (*p* < 0.05). The relative abundance of Treponema, Fibrobacter, Lachnospiraceae AC2044, and Prevotellaceae UCG-003 was higher in the HAY diet (*p* < 0.05), while the relative abundance of the Rikenellaceae RC9 gut group, Prevotellaceae UCG-004, and Ruminococcus were higher in FO diet (*p* < 0.05).

### 3.7. Relative DNA Quantification of Total Bacteria, Archaea, and Protozoans by qPCR

To complement the data obtained from sequencing bacteria and given the significance of archaea and protozoa in the gastrointestinal microbiota of horses, quantification using real-time PCR was conducted. The diets did not significantly affect (*p* ≥ 0.05) the population of archaea and protozoa in the fecal and cecum samples of horses (Table 6).

## 4. Discussion

It is well established that feeding horses different energy sources leads to significant changes in their hindgut microbiota, as shown in both fecal [2,39] and cecal samples [4]. Given that alterations in microbial composition are often associated with equine health disorders [15], this study evaluated the digestive parameters and microbiota of the cecum and feces of horses maintained on three different diets: a hay-based diet (HAY), a diet rich in starch and sugar (SS), and a diet rich in fiber and oil (FO).

Although dietary differences in fiber and starch content are expected to influence SCFA concentrations, the various energy sources used in this study did not alter the concentrations of SCFA or the (acetate/butyrate)/propionate ratio in cecal and fecal samples, consistent with the findings of Muhonen et al. [40]. This suggests that while the diets influenced the abundance of cecal and fecal microorganisms and the diversity of the fecal microbiota in healthy horses, these changes were not sufficient to significantly impact the fermentation process. Factors such as adaptation time to different energy sources may have contributed to the stability of SCFA levels. Warzecha et al. [4] observed variations in cecal pH and SCFA concentrations 6 h after feeding a high-concentrate diet compared to a low-concentrate diet, with these effects lasting up to 7 days.

Conversely, differences in the diets influenced the pH and buffering capacity, suggesting that variations in dietary fiber regimes and composition may affect the gastrointestinal tract [27,41]. Prolonged periods with hindgut pH values below 6.2 can disrupt fermentation and destabilize the hindgut microbiome [17], but the diets tested in this study did not reach these levels (cecum pH ≥ 7.18). The higher pH value observed in the HAY diet aligns with the microbiome associated with the degradation of slowly fermentable carbohydrates, which is essential for digestive health. The fecal pH values recorded in this study (6.14 to 6.70) were also within the normal range suggested in the literature for horses receiving high-fiber or high-concentrate diets [42]. Adequate foraging time is crucial for horses to meet fiber requirements, maintain gut fill, stimulate saliva production, and ensure proper buffering capacity [43]. However, our study found higher BC5 levels in fecal samples from horses on the SS diet and higher BC6 levels in cecal samples from those on the SS and FO diets compared with the HAY diet, which contrasts with findings reported by Luthersson and Nadeau [44].

In this study, the different dietary sources influenced the alpha diversity (Shannon index) in fecal samples, with the highest species diversity observed in the SS diet, followed by the FO and HAY diets. This suggests that concentrate-rich diets may promote bacterial growth. However, no differences in alpha diversity were found in cecum samples, which could be attributed to stable SCFA concentrations and consistent pH levels across the different energy sources. Warzecha et al. [4] and Sorensen et al. [45] linked the availability of soluble carbohydrates to increased SCFA concentrations and reduced pH, which can affect the survival of microbial populations and decrease species diversity. The lack of differences in diversity is a favorable outcome, suggesting that the diets are likely safe for the digestive health of horses [4,15,46,47]. In contrast, previous studies [48] reported no differences in alpha diversity in fecal samples but found higher alpha diversity in the cecum of horses fed a high-fiber compared to a high-starch diet, indicating a more stable microbiota and greater resistance to pathogens.

This study underscores the importance of cecal cannulation for accurately assessing digestive parameters and microbiota. Beta diversity analysis revealed significant differences between the microbial populations in fecal and cecal samples, aligning with Sorensen et al. [45], who reported variations in microbial diversity, SCFA concentrations, and pH between these sites. These findings suggest that fecal samples do not provide an accurate representation of the cecal environment. Notably, there is a marked discrepancy between the microbial populations of the upper gastrointestinal tract (stomach, jejunum, and ileum), which exhibit greater diversity both across regions, compared to the lower gastrointestinal tract (cecum and colon), where microbial differences are subtle. The high variability in the upper tract microbiota may be due to the introduction of new microbial populations through feed, while the lower variability in the lower tract microbiota could be attributed to slower passage rates and more stable microbial communities [49]. Such evidence demonstrates that different regions of the equine gastrointestinal tract have distinct microbial diversity profiles.

At the phylum level, the FO diet resulted in a lower relative abundance of Firmicutes, Fibrobactera, Desulfobacterota, and Spirochaetota in cecal samples, while Bacteirodota and Verrucomicrobiota were more abundant in both cecal and fecal samples. Bacteirodota, previously described as Bacteroidetes [50,51], has been positively associated with horse survival rates and linked to propionate production pathways [52]. It also plays a role in the later stages of plant fiber degradation, particularly in the breakdown of hemicellulosic polymers [53]. Verrucomicrobiota, on the other hand, is positively correlated with the expression of immune regulatory genes in the gastrointestinal tract of horses and supports the expression of the T-cell transcription regulatory factor Foxp3, indicating its potential role in promoting immunity [49,54].

As reported by Dougal et al. [2], fecal samples from horses fed a hay-based diet exhibited a higher abundance of the Fibrobacteraceae family, a finding that aligns with the results of the present study. However, while the authors observed a greater abundance of the Spirochaetaceae family in a fiber and oil diet, the present study found this family more abundant in the HAY diet. Notably, Fibrobacteraceae was the only family with a relative abundance greater than 1% that remained consistent across different collection sites, which may reflect its ability to persist under various environmental conditions and its reliance on a continuous supply of substrates [2]. In contrast, other families showed considerable variation between diets and collection sites.

The abundance of ASVs at the genus level provided a more accurate characterization of the effect of the HAY, SS, and FO diets on cecal and fecal microbiota. In the cecal environment, the SS diet was associated with a higher abundance of *Treponema*, *Desulfovibrio*, and the *Lachnospiraceae* AC2044 group. *Treponema* and *Lachnospiraceae* have been reported as more abundant in healthy horses, suggesting their importance in maintaining gastrointestinal health [55]. Additionally, *Treponema* was more prevalent in the HAY diet in fecal samples, consistent with its role in fiber degradation [48]. *Desulfovibrio* has been identified as a characteristic member of the mucosal microbiota in the cecum of healthy horses, distinct from the microbiota in the lumen and other regions of the gastrointestinal regions [49]. Microbial populations adhered to the mucosa can engage in pathways mediated by direct contact, such as the host immune system response [49]. Furthermore, the *Lachnospiraceae* AC2044 group, known for its role in fiber fermentation and butyrate production, has been linked to withdrawal behavior, which is common in animals kept in pens and more vulnerable to stress conditions [56,57].

The FO diet showed a higher abundance of *Prevotella*, *Prevotellaceae* UCG-003, *Akkermansia,* and *Anaerovibrio* in cecal samples. The genus *Prevotellaceae* UCG-003 is considered a key bacterium in the intestine of diarrhea-free piglets, contributing to the protection of the intestinal environment against pathogens, while the genus *Prevotella* has been associated with higher performance in both pigs [58] and horses [59]. *Anaerovibrio* is linked to fermentation processes and increases rapidly with the availability of hemicellulose; it is significantly affected by fasting time in fecal samples [60] and is also associated with lipid-rich horse diets, functioning as a lipolytic bacterium [61]. However, previous studies [4] have shown that the abundance of *Anaerovibrio* in cecal samples rises within 12 h following starch inclusion, contrasting with its increase observed with the FO diet in the present study. Finally, *Akkermansia* (phylum Verrucomicrobiota) has been correlated with improved health status in horses, supporting intestinal barrier function and reducing intestinal inflammation [54,62]; it has also been found in lower abundance from small intestinal colic [63].

In fecal samples, the SS diet showed a higher abundance of *Streptococcus*, a genus known as starch fermenters and lactic acid producers, which is often associated with intestinal acidosis [64]. *Streptococcus* is frequently linked to gastrointestinal disorders in horses [63,65] and is more abundant in low-performance horses [59]. Although the SS diet did significantly alter SCFA values, it was associated with an increase in populations related to the stress response, as indicated by the elevated levels of Streptococcus in feces. In contrast, the FO diet impact on the fecal microbiome was marked by a higher abundance of the *Rikenellaceae RC9 gut group*, a genus associated with horses fed forage and known for its ability to degrade structural carbohydrates [66]. Previous studies have reported higher levels of this genus in the cecum and colon of mice fed a high-fat diet and found positive correlations with propionate production in the cecum and acetate and butyrate production [67]. The observed increase in the *Ruminococcus* genus with the fiber and oil diet in this study aligns with the findings of Dougal et al. [2], who linked this genus to fat metabolism pathways.

In summary, the different energy sources provided to horses in a maintenance state did not significantly impact key gastrointestinal health parameters such as pH and SCFA concentrations. However, buffering capacity (BC) was affected, which may be linked to variations in microbial populations observed with each diet. While the long-term effects of these diets on horse health remain uncertain, the abundance of certain species suggests that the HAY and FO diets may be safer than the SS diet, as the SS diet increased genera associated with both immune responses and a higher risk of pathologies in horses.

While this study provides valuable insights that could inform dietary adjustments to enhance the performance and well-being of horses, several limitations should be acknowledged. First, the sample size was relatively small, comprising only five animals, and was further reduced by limited access to the cecum cannula and experimental conditions. This constraint may affect the generalizability of our findings to a larger horse population. Additionally, the use of specific PCR primers and 16S rRNA gene sequencing may introduce minor biases, as these methods can influence the amplification of certain microbial taxa over others, which could subtly affect the representation of microbial diversity and abundance [29,30]. The choice of bioinformatics pipeline, though standardized in this study, may also influence results, as differences in sequence quality filtering, amplicon sequence variants (ASVs), and taxonomic classification can impact the observed microbial community structure [68]. Future research with larger sample sizes and additional sequencing methods will be important to confirm and build on these findings, providing a clearer view of how diet affects equine gut health.

## 5. Conclusions

Diets based on different energy sources influence the abundance and diversity of cecal and fecal microbiota in healthy horses without significantly affecting archaea and protozoa populations or key gastrointestinal health parameters. Under the experimental conditions, these dietary adjustments did not disrupt fermentation processes or pose health risks, supporting the use of oil as a viable energy source to maintain intestinal health. However, further research is required to confirm these findings across broader equine populations and to evaluate potential long-term impacts on equine health and productivity.

## Figures and Tables

**Table 1:** Chemical composition of hay, starch and sugar (SS), and fiber and oil (FO) diets.

Item	Hay Diet	SS Diet	FO Diet
Dry matter (DM) (% as-fed)	93.6	92.3	93.2
Ash (% DM)	8.4	9.3	6.6
Crude protein (% DM)	12.9	13.9	13.7
Ether extract (% DM)	1.4	2.7	5.4
Neutral detergent fiber (% DM)	74.0	59.8	61.9
Acid detergent fiber (% DM)	39.8	31.1	34.2
Crude fiber (% DM)	36.9	28.4	31.5
Hemicellulose (% DM) ^1^	34.2	28.7	27.7
Starch (% DM)	0.1	6.7	2.3
Estimated digestible energy * (Mcal kg^−1^)	1.6	1.9	2.2
Crude energy (Mcal g^−1^)	4.2	4.0	4.0

^1^ Hemicellulose is calculated as follows: Hemicellulose = Neutral detergent fiber − Acid detergent fiber. * Calculated according to NRC (2007): Feed digestible energy = 4.07 − 0.55 × (Acid detergent fiber) and Hay digestible energy = 2.118 + (0.01218 × Crude protein) − (0.0093 × Acid detergent fiber) − [0.00383 × (Neutral detergent fiber-Acid detergent fiber)] + (0.04718 × Ether extract) − (0.0262 × Ash).

**Table 2:** PCR primers used for real-time qPCR assay.

Primer	5′→3′ Sequence	Amplicon Size	References
1114F (total bacteria)	CGGCAACGAGCGCAACCC	130 bp	Denman and McSweeney [35]
1275R (total bacteria)	CCATTGTAGCACGTGTGTAGCC		
958F (archaea)	AATTGGAKTCAACGCCGGR	142 bp	Poulsen et al. [36]
1100R (archaea)	TGGGTCTCGCTCGTTG		
316F (protozoa)	GCTTTCGWTGGTAGTGTATT	223 bp	Sylvester et al. [37]
539R (protozoa)	CTTGCCCTCYAATCGTWCT		

**Table 3:** Effect of diet on the SFCA concentration and the ratio of (acetate/butyrate)/propionate of cecal and fecal samples from healthy horses.

	HAY Diet	SS Diet ^1^	FO Diet ^2^	Diet (*p*-Value)
SCFA cecum (mmol/L) *				
Acetate	27.62 ± 2.69	28.56 ± 3.11	21.26 ± 3.81	0.4029
Propionate	9.82 ± 0.59	10.05 ± 0.68	7.37 ± 0.84	0.1605
Butyrate	2.69 ± 0.25	2.63 ± 0.28	1.65 ± 0.33	0.1059
Isobutyrate	0.19 ± 0.06	0.52 ± 0.07	0.31 ± 0.08	0.0576
Valerate	0.15 ± 0.03	0.26 ± 0.04	0.10 ± 0.05	0.1467
Isovalerate	0.15 ± 0.04	0.34 ± 0.04	0.15 ± 0.05	0.0805
Total SCFA	40.62 ± 3.53	42.36 ± 4.07	30.84 ± 4.99	0.3052
(acetate/butyrate)/propionate	3.05 ± 0.13	3.11 ± 0.15	3.10 ± 0.18	0.9459
SCFA feces (mmol/L) *				
Acetate	6.62 ± 0.89	7.88 ± 0.90	7.32 ± 0.97	0.4400
Propionate	2.75 ± 0.29	3.26 ± 0.29	3.14 ± 0.32	0.3822
Butyrate	0.56 ± 0.10	0.71 ± 0.11	0.63 ± 0.11	0.3411
Isobutyrate	0.21 ± 0.03	0.25 ± 0.03	0.22 ± 0.03	0.5022
Valerate				
Isovalerate	0.12 ± 0.05	0.25 ± 0.07	0.35 ± 0.13	0.3383
Total SCFA	10.26 ± 1.34	12.35 ± 1.34	11.65 ± 1.46	0.4141
(acetate/butyrate)/propionate	2.61 ± 0.11	2.57 ± 0.11	2.49 ± 0.12	0.6127

* Mean estimate and standard deviation. ^1^ Starch and sugar diet; ^2^ Fiber and oil diet.

**Table 4:** Effect of diet on the pH and buffering capacity (BC) of cecal and fecal samples from healthy horses.

	HAY Diet	SS Diet ^1^	FO Diet ^2^	Diet (*p*-Value)
pH				
Cecal	7.18 ± 0.10	7.45 ± 0.11	7.60 ± 0.14	0.1454
Fecal	6.70 ± 0.10 ^a^	6.22 ± 0.10 ^b^	6.14 ± 0.11 ^b^	0.0052
BC5 (mmol/L)				
Cecal	68.04 ± 5.04	70.93 ± 5.49	66.89 ± 6.19	0.7513
Fecal	19.81 ± 1.80 ^b^	25.99 ± 1.80 ^a^	17.65 ± 1.97 ^b^	0.0150
BC6 (mmol/L)				
Cecal	29.37 ± 2.48 ^b^	36.94 ± 2.67 ^a^	41.87 ± 2.96 ^a^	0.0301
Fecal	6.93 ± 0.76	5.37 ± 0.76	3.66 ± 0.85	0.0660

^a,b^ Means in a row followed by different letters are significantly different from each other (*p* < 0.05). ^1^ Starch and sugar diet; ^2^ Fiber and oil diet.

**Table 5:** Effect of diet on the Shannon diversity index and Chao1 richness of cecal and fecal samples from healthy horses.

Index	Hay Diet		SS Diet ^1^		FO Diet ^2^		Diet (*p*-Value)
	Mean	SE ^3^	Mean	SE ^3^	Mean	SE ^3^	
Cecal samples							
Shannon index	3.18	0.07	3.22	0.08	2.97	0.10	0.2700
Chao1	159.03	2.28	161.28	2.63	158.52	3.22	0.7662
Fecal samples							
Shannon index	3.40 ^b^	0.03	3.58 ^a^	0.03	3.48 ^ab^	0.04	0.0123
Chao1	172.74	6.03	174.04	6.03	168.95	6.73	0.8350

^a,b^ Means in a row followed by the same letters are not significantly different from each other (*p* > 0.05). ^1^ Starch and sugar diet; ^2^ Fiber and oil diet. ^3^ SE, standard error. Total of samples: HAY (5 fecal, 4 cecal), SS (5 fecal, 3 cecal), FO (4 fecal, 2 cecal); variations due to cecum access loss and one mortality.

**Table 6:** Relative quantification of archaeal and protozoan rDNA to total bacteria using qPCR in cecal and fecal samples from healthy horses.

Organism	Hay Diet		SS Diet ^1^		FO Diet ^2^	
	Mean	SE ^3^	Mean	SE ^3^	Mean	SE ^3^
Cecal samples						
Archaea	2.02	0.07	1.95	0.07	1.80	0.08
Protozoa	2.11	0.07	2.08	0.07	2.03	0.08
Fecal samples						
Archaea	2.26	0.09	2.04	0.11	2.08	0.13
Protozoa	1.91	0.09	1.94	0.11	1.64	0.13

^1^ Starch and sugar diet; ^2^ Fiber and oil diet. ^3^ SE, standard error. Total of samples: HAY (5 fecal, 4 cecal), SS (5 fecal, 3 cecal), FO (4 fecal, 2 cecal); variations due to cecum access loss and one mortality.

## Data Availability

The original contributions presented in this study are included in the article/Supplementary Materials. Further inquiries can be directed to the corresponding author.

## Supplementary Materials

The following supporting information can be downloaded at https://www.mdpi.com/article/10.3390/ani14233494/s1, Figure S1: Rarefaction curves for microbial populations in cecal and fecal samples from healthy horses. Table S1: Feed intake per meal and per kilogram of body weight. Table S2: Molar percentages of individual short-chain fatty acids (SCFAs).
