# Supplementary figures and images for: Dietary Energy Sources Affect Cecal and Fecal Microbiota of Healthy Horses
Source: Animals (Basel). 2024 Dec 3;14(23):3494. doi: 10.3390/ani14233494 (PMC11639918; doi:10.3390/ani14233494)

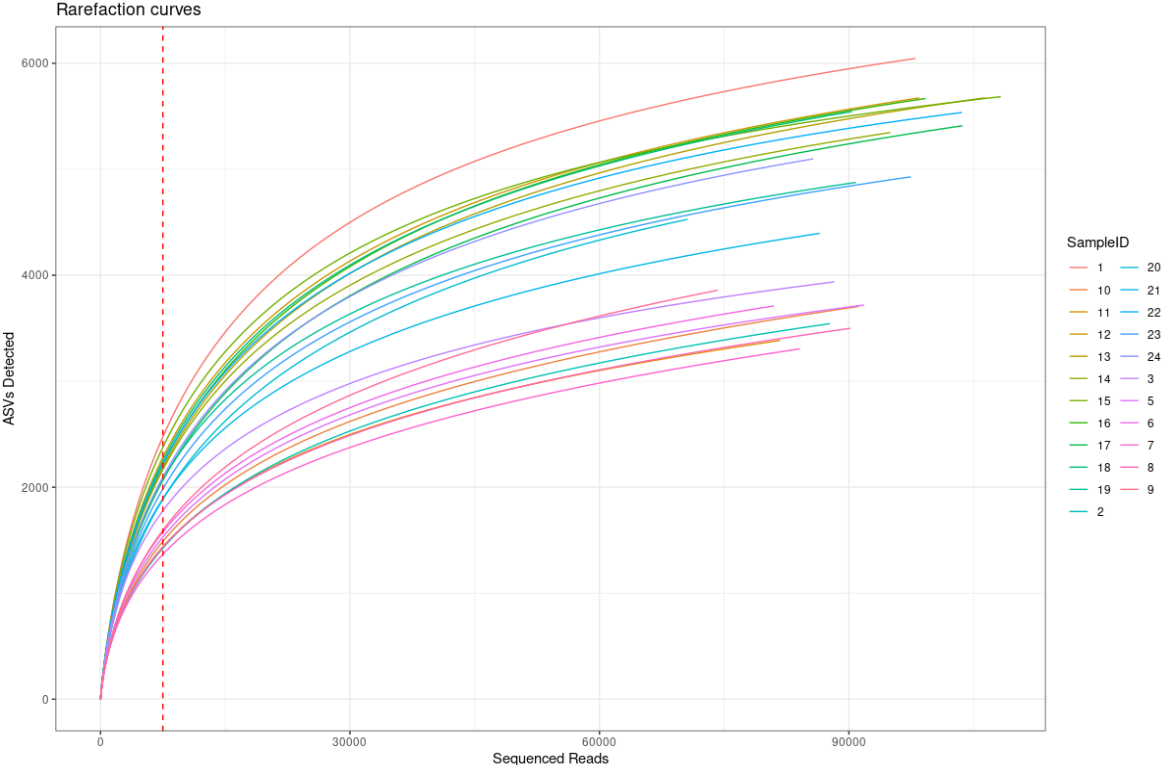

Supplement: Supplementary file 1 [file animals-14-03494-s001.zip › animals-3278555-supplementary.tif]
